# Encysted Odyssey: A Clinical and Pictorial Analysis of Hydatid Cysts From Head to Toe

**DOI:** 10.7759/cureus.61180

**Published:** 2024-05-27

**Authors:** Sankeerth Kendyala, Ramakrishna Narayanan

**Affiliations:** 1 Radiology, Nizam's Institute of Medical Sciences, Hyderabad, IND

**Keywords:** radiology, cross-sectional imaging, diagnostic imaging features, echinococcus granulosus, hydatid disease, cystic echinococcosis

## Abstract

Introduction: Cystic echinococcosis, a zoonotic disease caused by the larval form of *Echinococcus granulosus*, predominantly affects the liver and lungs, with humans acting as accidental hosts.

Methods: Our retrospective study at the Department of Radiology and Imageology, Nizam’s Institute of Medical Sciences, included 187 histopathologically or serologically proven cases. The mean age of presentation was 49.4 years.

Results: Liver involvement was most prevalent, accounting for 83.4% (n=156) of cases, followed by sporadic involvement of other organs such as the mesentery, spleen, pancreas, thalamus, kidney, lung, spine, and omentum. Characteristic diagnostic features observed on imaging included peripheral calcifications in 33% of cases, internal septations in 25% (n=47), dense calcifications in 15% (n=29), daughter cysts in 6% (n=11), and floating membranes in 5% (n=10). Among hepatic lesions, 90% (n=141) were showing involvement of a single lobe. Notably, 78% (n=110) of lesions were limited to the right lobe, 21% (n=30) to the left lobe, and 1% (n=1) to the caudate lobe. The most affected hepatic segment was segment VIII, while the least common was segment I (caudate lobe). Complications were identified in 13% (n=25) of cases of hepatic hydatidosis.

Conclusions: The findings of our study emphasize the systemic nature of *E. granulosus *infection which can affect various organs in the body. It also illustrates the invaluable insights imaging provides for timely and accurate diagnosis of hydatid disease.

## Introduction

Cystic Echinococcosis is a zoonotic disease caused by the larval form of *Echinococcus granulosus*. These echinococcal worms primarily reside in dogs, while sheep serve as the most common intermediate hosts. Humans typically act as accidental hosts. Infection can occur through the consumption of food contaminated with dog faeces or direct contact with infected dogs. The liver is the most commonly affected organ, accounting for 63% of cases, followed by the lungs (25%), muscles (5%), bones (3%), kidneys (2%), spleen (1%), and other areas (1%) [1]. These affected sites often exhibit identifiable imaging characteristics. It can cause various complications like intrahepatic biliary radical dilatation (IHBRD), dissemination, rupture, peritoneal spreading, and involvement of the portal vein.

Clinical symptoms can vary significantly based on several factors: (a) the organ affected, (b) the size and location of the cyst, (c) interactions between growing cysts and adjacent organs, and (d) complications stemming from cyst rupture [2-4]. A timely pre-operative diagnosis of cystic echinococcosis is essential to prevent anaphylactic reactions or local recurrence. The use of cross-sectional imaging techniques, such as computed tomography (CT) and magnetic resonance imaging (MRI), is crucial for accurately characterizing the disease and identifying associated complications, particularly when the disease appears in unusual locations.

## Materials and methods

The aims and objectives of this study were threefold. Firstly, we aimed to comprehensively evaluate the distribution of hydatid disease throughout the body. Secondly, we aimed to elucidate the particular patterns and manifestations of hepatic hydatid disease. Lastly, we aimed to delineate the characteristic radiologic findings of hydatid disease and its associated complications.

We conducted a retrospective observational study within the Department of Radiology at Nizam’s Institute of Medical Sciences, with a sample of 187 imaging and histopathological or serological proven hydatid disease patients from December 2018 to August 2023. The search for patients to include in the study was done using a radiology information system. The search was conducted for patients aged 18 years or older, who were reported to have hydatid disease based on imaging findings on cross-sectional imaging. Initially, 246 patients were included, out of which 42 patients were excluded due to the absence of histopathological or serological confirmation, and a further 17 were excluded due to suboptimal cross-sectional imaging, leading to a final sample of 187 patients. The images of examinations were retrieved by the local search and were reviewed comprehensively to assess for the distribution of hydatid cysts, identify characteristic imaging features, and determine any associated complications. Cross-sectional imaging for all cases was carried out using either CT, MRI, or both modalities. The diagnosis of these cases was confirmed either through histopathological examination or serological analysis. Informed consent was taken from all the participants in the development data set and to publish the images if required. Findings were summarized using descriptive statistics. Analysis was performed using Microsoft Excel (version 2018; Microsoft® Corp., Redmond, WA, USA).

Demographic data such as age distribution, gender prevalence, and dietary habits were evaluated. On imaging, a preliminary assessment of the number of lesions and distribution of lesions, i.e. hepatic or extrahepatic disease, was done. Characteristic diagnostic features, such as peripheral calcifications, internal septations, dense calcifications, daughter cysts, floating membranes, etc., were examined. Hepatic hydatid lesions were further assessed for multiplicity, lobar and segmental distribution of lesions, and associated complications.

Representative images from varied distributions of hydatid disease are illustrated to demonstrate characteristic imaging features and associated complications across varied organ distributions.

## Results

The average age of presentation for hydatid disease was 49.4 years. Our demographic breakdown showed that males comprised 55% and females 45% of the study population. Dietary habits revealed that 90% of patients consumed a mixed diet, while 10% were vegetarians.

**Figure 1:**
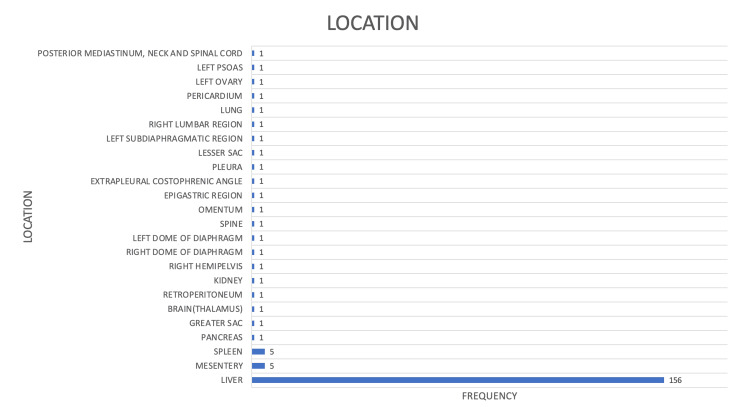
Varied organ distribution in hydatid disease

**Figure 2:**
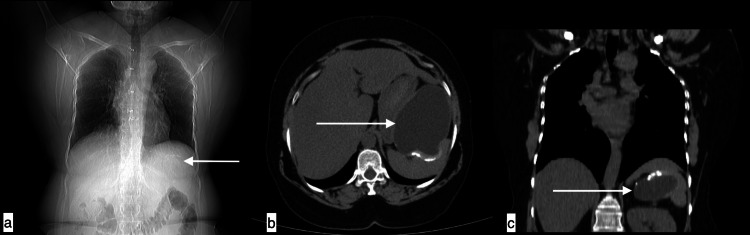
Mesenteric hydatid cyst A 56-year-old male with mesenteric hydatid cyst presented with left upper quadrant pain increasing with exertion for four years. Topogram image (a), axial image (b), and coronal image (c) of non-contrast CT chest revealed a well-defined low attenuating fluid density consistent with thick-walled cystic lesion with dense peripheral calcifications noted in the left upper quadrant, which is seen arising from mesentery, causing indentation over stomach medially and spleen posteriorly.

**Figure 3:**
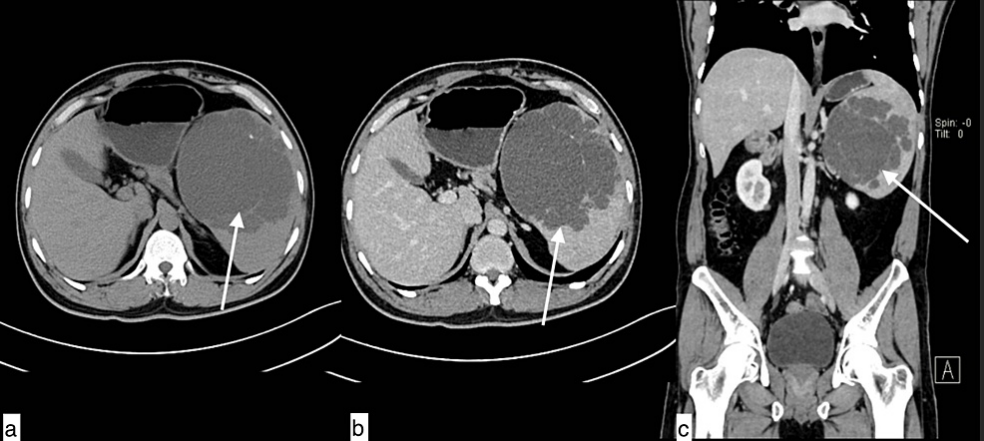
Splenic hydatid cyst A 40-year-old male with a splenic hydatid cyst presented with pain and swelling in the left hypochondrium. Axial plain (a), axial and coronal post-contrast (b and c) images showing a hypodense cystic lesion with ill-defined margins in splenic parenchyma showing thin intralesional septations with fine calcifications. The lesion is indenting on the fundus of the stomach and displacing the left kidney.

**Figure 4:**
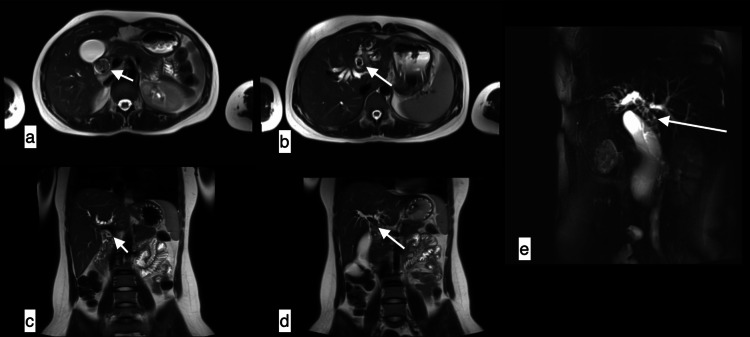
Biliary hydatid cyst A 23-year-old female with a biliary hydatid cyst presented with fever, pain abdomen, jaundice, and deranged LFTs. Axial (a, b) and coronal (c, d, e) MRCP images showing T2 hypointensity in the dilated left hepatic duct, CHD, CBD with curvilinear soft tissue signal intensity contents within the CBD and IHBRD. LFTs: liver function tests; MRCP: magnetic resonance cholangiopancreatography; CHD: common hepatic duct; CBD: common bile duct; IHBRD: intrahepatic biliary radical dilatation

**Figure 5:**
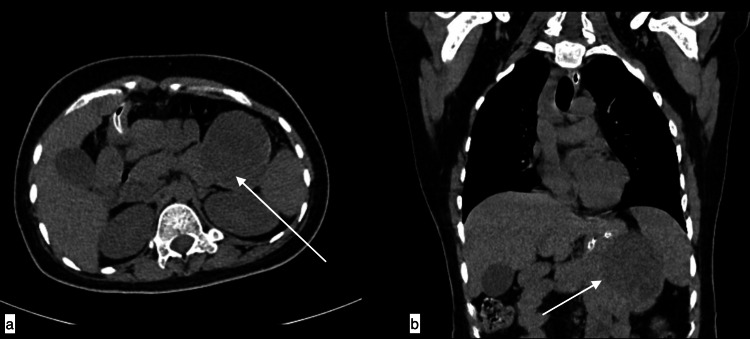
Pancreatic hydatid cyst A 24-year-old female with pancreatic hydatid cyst presented with fever, cough, and shortness of breath for two months. Axial (a) and coronal (b) non-contrast CT abdomen revealed a well defined thin walled hypodense lesion of fluid attenuation with wall calcification measuring 6.5x6x6 cm noted along the distal body and tail of pancreas extending into lesser sac, laterally abutting the splenic hilum with few internal septations.

**Figure 6:**
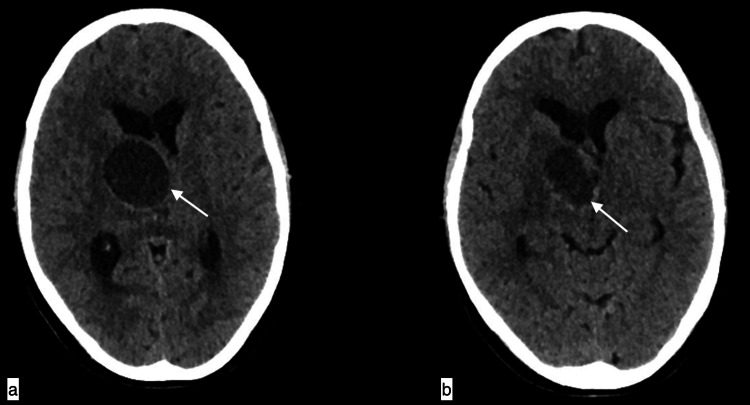
Intracranial thalamic hydatid cyst A 70-year-old female with an intracranial thalamic hydatid cyst presented with headache, left-sided weakness, and vomiting for three months. Axial CT brain showing a well-defined hypodense lesion of +16HU with hyperdense rim involving the right thalamus causing a mass effect in the form of midline shift towards left and compression of bilateral lateral ventricles and the foramen of Monro resulting in hydrocephalus.

**Figure 7:**
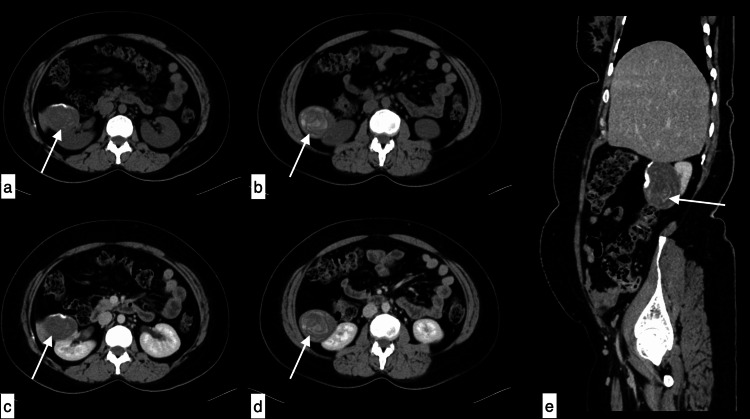
Renal hydatid cyst A 45-year-old male with a renal hydatid cyst presented with pain in the right hypochondrium and right flank. Axial non-contrast (a, b), axial (c, d) and sagittal (e) post-contrast images show a large well defined predominantly exophytic heterogeneous hypodense lesion with focal peripheral wall calcifications with dense linear contents within noted arising from interpolar of right kidney, showing no post-contrast enhancement.

**Figure 8:**
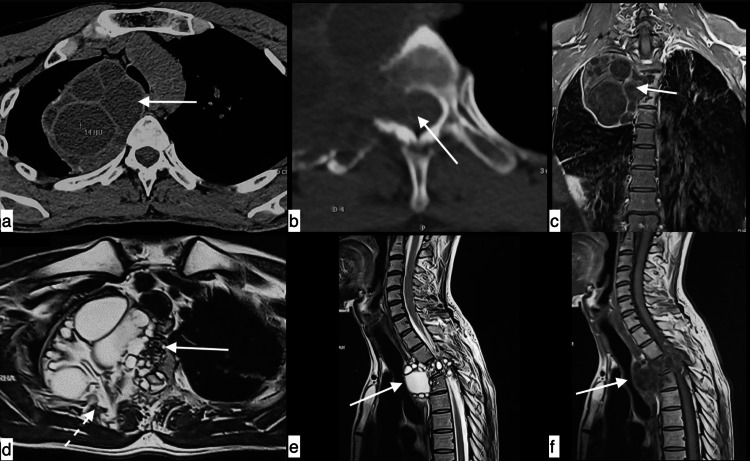
Spinal hydatid cyst A 36-year-old male with a spinal hydatid cyst presenting with cough, fever, and back pain, Axial plain CT (a and b) and coronal (c), axial (d), and sagittal MRI (e and f) showing lytic lesions with well-defined cystic lesions with daughter cysts in spinous process, right transverse process and body of D3, D4 vertebral bodies. The lesion is scalloping the posterior end of the second rib on the right side (dotted arrow) causing bone expansion and projecting into the canal with narrowing and impingement of the cord.

**Figure 9:**
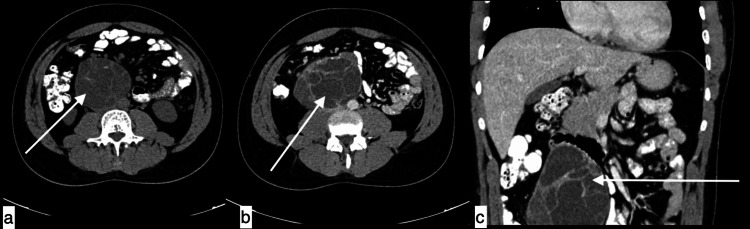
Retroperitoneal hydatid cyst A 26-year-old male with a retroperitoneal hydatid cyst presented with right flank pain and discomfort. Axial plain (a), axial post-contrast (b) and reformatted coronal post-contrast (c) CT showing a large well defined heterogeneously hypodense solid cystic multiloculated lesion in retroperitoneum with enhancing walls and internal septations with non-enhancing hypodense exophytic component, displacing bowel loops anteriorly, abutting and compressing IVC and aorta posteriorly with contact area >180 degrees. IVC: inferior vena cava

**Figure 10:**
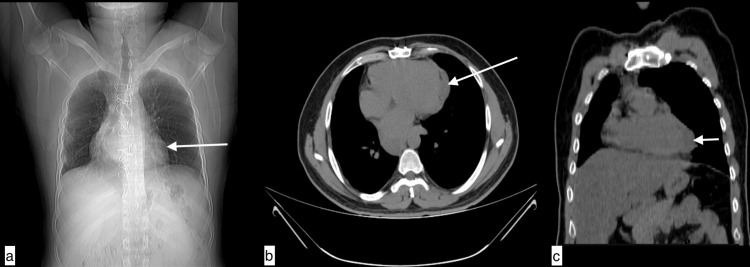
Pericardial hydatid cyst A 32-year-old male with a pericardial hydatid cyst presented with fever, dry cough, chest pain, and severe exertional dyspnea for two weeks. Topogram image (a), axial image (b), and coronal image (c) of non-contrast CT chest revealed a well-defined hypodense (+30 to +36HU) lesion in the right para oesophagal region extending into the middle mediastinum and along the pericardium with loss of fat planes with underlying myocardium and adjacent major vessels.

**Figure 11:**
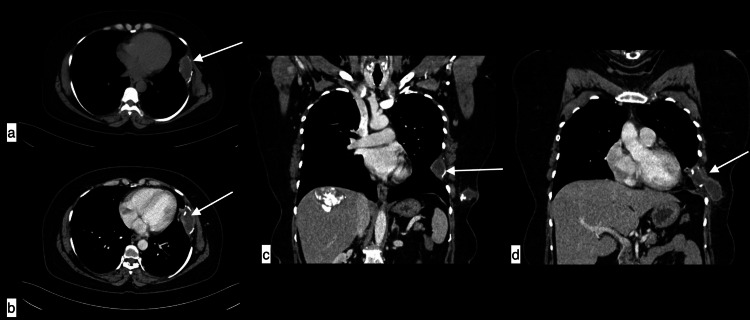
Hydatid cyst involving rib A 38-year-old male with a costal hydatid cyst presented with pain and swelling in the left chest wall for three years. Axial plain (a), axial post-contrast (b), and coronal post-contrast (c and d) images revealed a lobulated multilocular cystic lesion with few peripheral calcifications, located on the lateral aspect of the left seventh rib, associated with significant deformation and destruction of the internal compact layer of the rib with exophytic component seen extending into adjacent subcutaneous fat. Additionally, a calcified hepatic hydatid cyst is noted.

**Figure 12:**
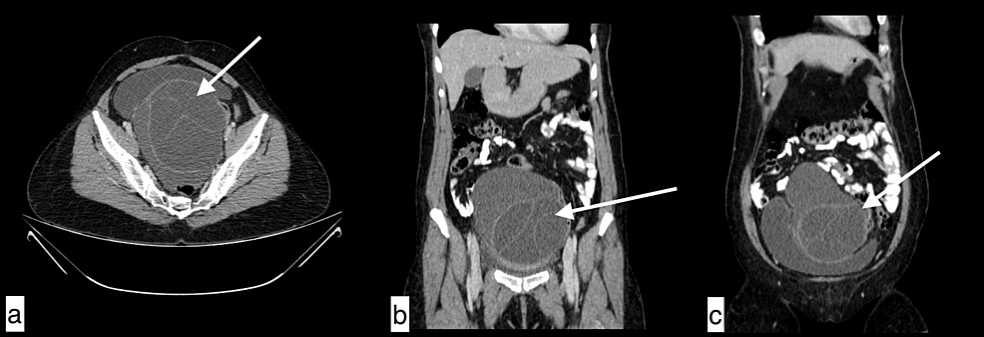
Ovarian hydatid cyst A 40-year-old female with ovarian hydatid cyst presented with pelvic pain for three months. Axial (a) and coronal post-contrast (b and c) images revealed a large well-defined thin walled multi-loculated cystic lesion (+10HU) with multiple thin internal septations measuring 12.1x9.7x11.6 cm with thin hyperdense wall noted in pelvis more on left adnexa causing compression and displacement of bladder antero-superiorly with no enhancement on post contrast. The left ovary was not seen separately.

In 95% of cases, focal lesions were detected, whereas 5% exhibited multifocal lesions. The liver was the most frequently affected organ, accounting for 83.4% of cases (Figure 1), followed by involvement of the mesentery (Figure 2) and spleen at 2.7% (Figure 3). Isolated cases were also observed affecting the biliary tree (Figure 4), pancreas (Figure 5), brain (thalamus) (Figure 6), kidney (Figure 7), lung, spine (Figure 8), retroperitoneum (Figure 9), pericardium (Figure 10), rib (Figure 11) and ovary (Figure 12).

**Figure 13:**
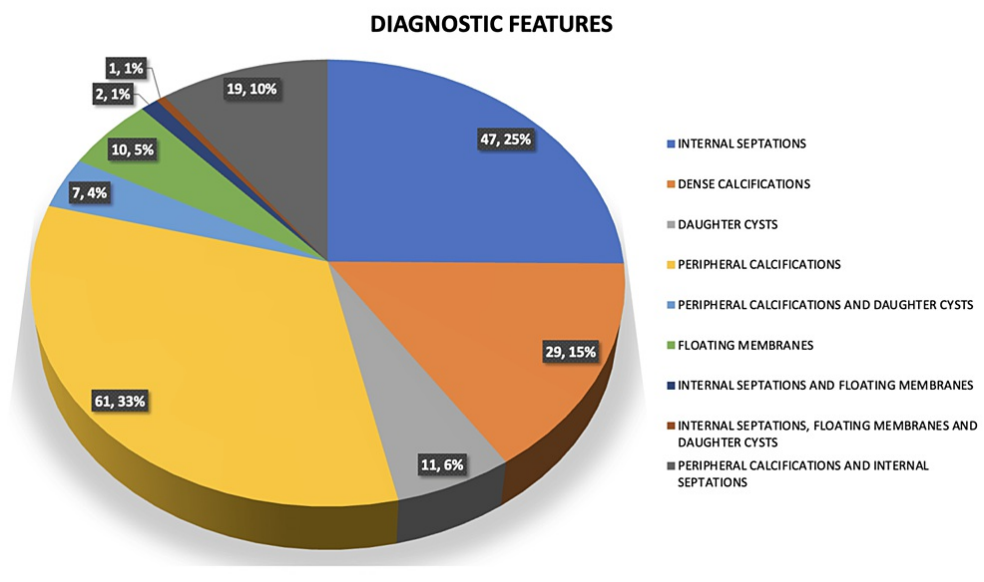
Frequency of distribution of diagnostic characteristics in various hydatid cysts in our demographic

The characteristic diagnostic features included peripheral calcifications in 33% of cases, internal septations in 25%, dense calcifications in 15%, daughter cysts in 6%, and floating membranes in 5%. Features overlapped in the remaining cases (Figure 13).

The average lesion size was 6.66 cm. The smallest lesion measured 1.5 cm in its longest dimension, while the largest lesion spanned 22 cm.

**Figure 14:**
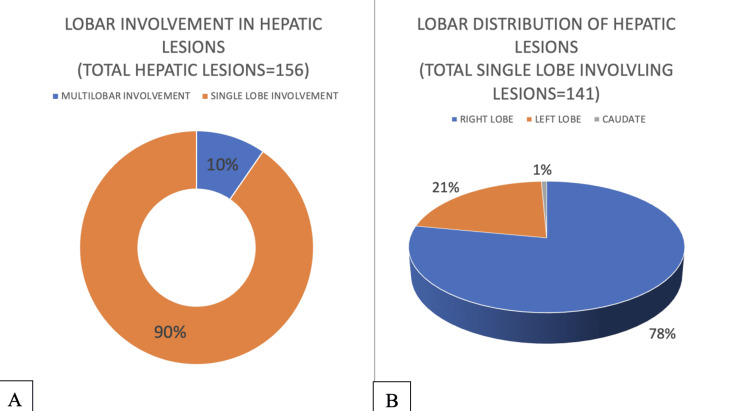
Figure 14A showing lobar involvement in hepatic hydatid cysts and Figure 14B showing lobar distribution of hepatic hydatid cysts

**Figure 15:**
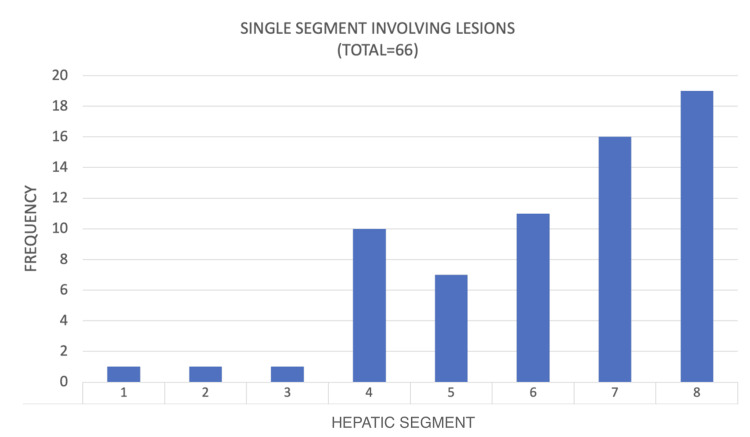
Frequency of single segment involving hepatic hydatid cysts

Regarding hepatic lesions, 89% were solitary, and 11% were multiple. A single lobe was involved in 90% of cases, while multilobar involvement was observed in 10%. Specifically, 78% of lesions were restricted to the right lobe, 21% to the left lobe, and 1% to the caudate lobe (Figure 14). Segment VIII was the most commonly affected hepatic segment, while segments III, II, and I were the least involved (Figure 15).

**Figure 16:**
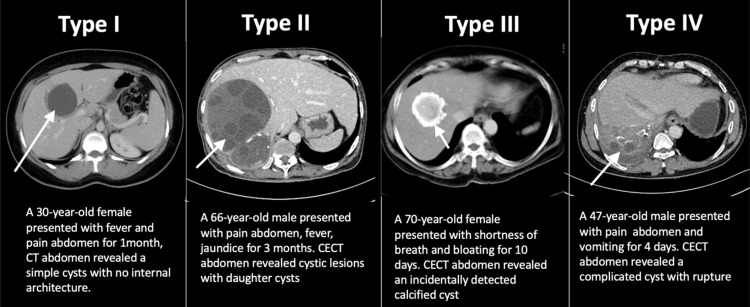
Types of hepatic hydatid cysts with imaging features CECT: contrast-enhanced computed tomography

Based on medical imaging morphology, hepatic hydatid cysts were categorized into four types: Type III accounted for 46%, Type II for 36%, Type IV for 13%, and Type I for 5% (Figure 16).

**Figure 17:**
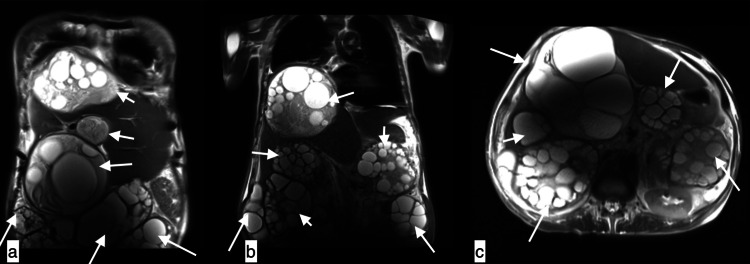
Peritoneal hydatidosis A 52-year-old male with peritoneal hydatidosis presented with progressively increasing abdominal distension, weight loss, and anorexia. Coronal (a,b) and axial (c) MRI abdomen showing multiple cystic lesions with peripherally arranged daughter cysts of varying sizes in the right lobe of the liver, subhepatic space, and visualized peritoneal cavity causing superior displacement of the diaphragm and causing compression of the right lower lobe. Cysts in the peritoneal cavity are compressing the pancreatic head and tail, bilateral kidneys with right minimal pleural effusion.

**Figure 18:**
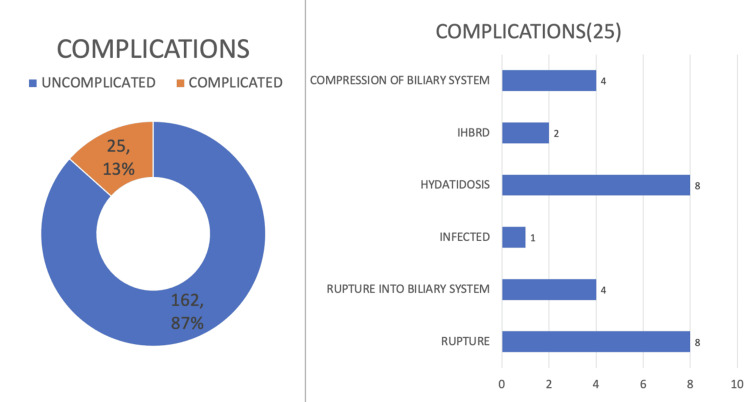
Complications of hydatid cysts IHBRD: intrahepatic biliary radical dilatation

Among hepatic hydatid cysts, 87% were uncomplicated, while 13% (25 cases) presented with complications. The most common complications were hydatidosis (Figure 17) and rupture, each accounting for 32%. This was followed by rupture into the biliary tree and biliary tree compression at 16%, IHBRD at 0.8%, and infection at 0.04% (Figure 18).

## Discussion

Hydatid disease is a zoonotic infection caused by tapeworms belonging to the *Echinococcus *genus. While dogs act as definitive hosts transmitting the disease, sheep frequently serve as primary intermediary carriers. Humans, however, are incidental, unintended hosts.

Within the intestines of their definitive hosts, hundreds to thousands of 3- to 7-mm-long *Echinococcus *adult worms develop. The final segment (or proglottid) of each worm matures to produce eggs, which are then excreted in the dog's faeces into the environment. Subsequently, either humans or intermediate hosts ingest these eggs. Once ingested, the eggs hatch in the intestine, releasing oncospheres. These oncospheres traverse the portal and lymphatic vessels, typically settling in the liver where they develop into larvae known as metacestodes or hydatid cysts. Less commonly, they may migrate to the lungs, brain, bones, or other organs of the human or intermediate host. Among the 187 cases studied, 85 were female and 102 were male. This contrasts with a study by Rao SS et al. [5], which reported a female predominance.

Our study found that the most commonly affected age group was between 50 and 60 years. This finding diverges from the research of Papadimitriou J et al. [6], which indicated that while hydatid cysts can manifest at any age, they are more prevalent in younger individuals. The age distribution observed in our study may be influenced by the absence of a pediatric department in our hospital, leading to a potential selection bias (Berksonian bias).

In our demographic, 90% of individuals adhere to a diverse diet, while the remaining 10% are vegetarians. The transmission of the infection can occur through various means, including direct contact with infected dogs, exposure to faeces containing eggs, and handling contaminated plants and soil - often through direct hand-to-mouth contact. Additionally, the ingestion of raw or improperly washed vegetables, salads, fruits, and contaminated water can lead to egg ingestion, further propagating the disease.

The liver emerged as the most frequently affected organ in our study, followed by the mesentery and spleen. Singular cases were also identified in organs such as the pancreas, thalamus, kidney, lung, spine, and omentum.

**Figure 19:**
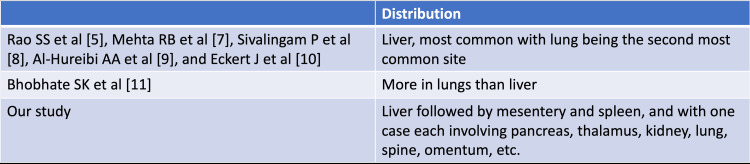
Comparison of organ distribution of cystic echinococcosis with previous studies

Previous studies by Rao SS et al. [5], Mehta RB et al. [7], Sivalingam P et al. [8], Al-Hureibi AA et al. [9], and Eckert J et al. [10] collectively underscored the liver as the predominant site, with the lungs ranking second. In stark contrast, Bhobhate SK et al. [11]reported a higher incidence in the lungs than in the liver. Our findings deviated from this trend, highlighting the liver as the primary site while showing varied involvement of other organs (Figure 19).

Spleen involvement occurs in 2.5-5.8% of cases, ranking third after liver and lung involvement [12]. While splenic hydatidosis is rare on its own, it often accompanies liver involvement, typically due to cyst rupture and subsequent spread throughout the body or abdomen. Common symptoms include an enlarged spleen, upper abdominal pain, and fever [13]. Imaging of splenic cysts mirrors that of liver cysts, often manifesting as single lesions with discernible calcifications on CT scans.

Spinal cord involvement is rare, accounting for less than 1% of all cases [14]. It predominantly affects the thoracic, lumbar, sacral, and cervical regions, with the thoracic and lumbar areas being most commonly affected. There are five types of cystic echinococcosis involving the spinal cord, with vertebral and paravertebral types being the most prevalent [14,15]. These types often exhibit multiple cysts on imaging. Contrast-enhanced imaging typically shows no enhancement, and calcification is infrequently observed in cases of spinal cord involvement [14].

Peritoneal-retroperitoneal cystic echinococcosis often arises from the rupture of a hepatic cyst or after surgical interventions involving these cysts. Primary involvement in this region is exceedingly rare [16]. Most individuals with cystic echinococcosis experience varied symptoms that are not exclusive to the condition. The manifestation of symptoms depends on factors such as the affected organs, cyst location within those organs, cyst size, and interaction with adjacent structures [16]. Imaging findings for peritoneal-retroperitoneal cystic echinococcosis resemble those of cystic echinococcosis in other organs. Approximately 5-14% of patients with liver hydatid cysts also develop peritoneal cysts [17,18]. The number and size of these cysts can vary significantly. Rare complications, like pelvic vein congestion due to pressure from a sizable peritoneal cyst, can also manifest [19].

In our study, hepatic hydatid cysts comprised 83.4% of the total distribution of hydatid cysts. Regarding intrahepatic distribution, we found that 78% of the lesions were located in the right lobe of the liver, 21% in the left lobe, and 1% in the caudate lobe. Similarly, Papadimitriou J et al. [6] reported that 85% of the cysts were situated in the right lobe of the liver, with only 15% in the left lobe. The predominance of the right lobe can likely be attributed to the straighter course of the right branch of the portal vein.

Liver echinococcosis accounts for 60-75% of cases, with 80% of hydatid cysts located in the right hepatic lobe [20,21]. Clinical symptoms typically manifest when a liver cyst exceeds 10 cm in diameter or occupies more than 70% of the liver's volume [22]. Patients with symptomatic liver cysts from cystic echinococcosis commonly report appetite loss and upper abdominal pain. Compression of the bile ducts can result in jaundice. Upon examination, doctors might observe abdominal swelling, a mass resembling a tumour, and an enlarged liver. Cyst rupture can occur spontaneously due to increased internal cyst pressure or as a consequence of surgical intervention or trauma. Rupture is reported in approximately 3-3.2% of patients with liver hydatid cysts [23]. Additional complications may encompass cyst infection, fever, hives (urticaria), elevated eosinophil count (eosinophilia), and in rare instances, severe allergic reactions (anaphylaxis) [24].

**Figure 20:**
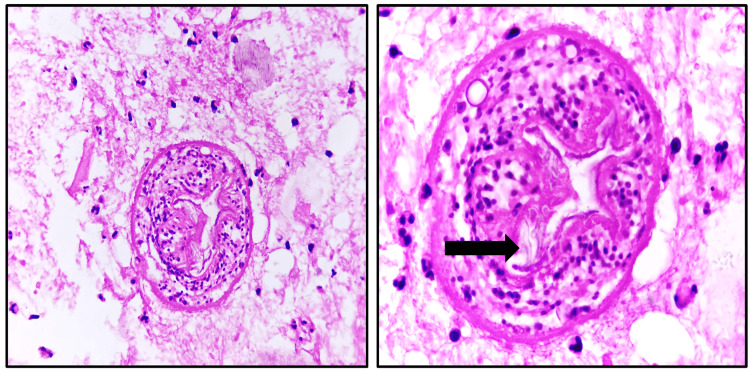
Biopsy specimen showing daughter cyst with inflammatory infiltrate

Our findings indicated that 46% of hepatic hydatid cysts were classified as Type III, 36% as Type II, 13% as Type IV, and 5% as Type I. Although Type II hydatid cysts are diagnostic of hydatid cysts due to the presence of daughter cysts (Figure 20) and membranes, our study revealed that Type III hydatid cysts containing calcifications were predominant in our demographic, followed by Type II, Type IV, and Type I cysts.

Hydatid disease can be classified into four types based on its appearance in radiological imaging [25]:

Type I: simple cyst with non-internal architecture

A Type I cyst appears clear and smooth on ultrasound (US), often presenting as a distinct, echo-free mass. Occasionally, it might contain hydatid sand or partitions. These cysts resemble the early stage of the parasite [26]. Distinguishing a single Type I cyst from a regular cyst using the US can be challenging. In CT, a Type I cyst resembles a clear mass akin to water. MRIs of hydatid cysts bear a resemblance to basic liver cysts, typically appearing as dark areas on T1-weighted images and very bright areas on T2-weighted images. A characteristic feature called the "rim sign" [27,28], visible as a low-intensity rim, is more prominent on T2-weighted images. This "rim sign" represents the area surrounding the cyst, known as the pericyst.

Type II: cyst with daughter cysts and matrix

Daughter cysts are contained within the mother cyst, typically arranged peripherally [26,29]. Floating membranes or vesicles may also be observed within the cyst. Multiple daughter cysts together resemble an echogenic solid lesion.

Type II can present as a well-defined fluid collection exhibiting a honeycomb pattern with multiple septa representing the walls of the daughter cysts, forming a “rosette” appearance [28]. Peripheral calcification involving the pericyst may occur and is easily discernible in CT images as a curvilinear or ring-like structure. CT can differentiate the mother cyst, as the average density attenuation of the mother cyst is higher than that of the daughter cysts. In MRI, daughter cysts may appear hypointense or isointense relative to the maternal matrix on both T1 and T2-weighted images [27,28].

Type III: calcified cyst

Type III lesions are characterized by dead cysts with complete calcification. On US, calcified cysts exhibit strong posterior shadowing; on CT, they appear as round hyperattenuating areas; and on MRI, they manifest as hypointense areas.

Type IV: complicated cyst

Complications associated with hydatid disease encompass rupture and superinfection, which can occur in both Type I and Type II. CT and MRI play pivotal roles in identifying these complications, such as cyst rupture and infection.

In our study, complications associated with hepatic hydatid cysts included hydatidosis, cyst rupture, compression of the biliary tree, rupture into the biliary tree, and IHBRD. A minority of cases also exhibited infections. The mesentery and spleen demonstrated involvement but at a lesser frequency, with fewer complications reported. Additionally, isolated cases affecting organs such as the pancreas, thalamus, kidney, lung, spine, and omentum were noted, each presenting with specific complications.

Hydatid cysts can cause many complications. They can cause a mass effect, which occurs when a cyst grows large enough to compress nearby bile ducts, leading to their dilation or even perforation. Another concern is the rupture of the cyst, which can occur internally, communicating with the biliary ducts, or externally, resulting in the spread of cystic contents into the peritoneal cavity, causing disseminated disease. Infections are also a risk, particularly in cases of ruptured cysts, as they can facilitate bacterial entry into the cyst. Detecting air within the cyst cavity, visible as thickening and enhanced walls on contrast-enhanced CT scans and MRIs, can serve as an indicator of infection. Additionally, there's the possibility of exophytic growth, where cysts extend beyond the liver's surface, migrating to the lung, mediastinum, or peritoneal cavity via different routes. Peritoneal seeding, often a consequence of prior hepatic hydatid disease or cyst rupture, is another concern, necessitating diagnostic imaging with CT scans and MRIs to identify peritoneal hydatid cysts accurately.

In contrast, Eckert J et al. [20] reported varied distributions of cases and complications across different organs. They identified the liver as the predominant site of involvement. Complications listed in their study included liver abscess, portal hypertension, inferior vena cava compression and thrombosis, Budd-Chiari syndrome, cyst rupture, peritoneal spread, biliary peritonitis, cholangitis, and pancreatitis. While lung involvement was less frequent, it presented with complications like biliptysis, pneumothorax, lung abscess, eosinophilic pneumonia, and parasitic lung embolism. Kidney involvement, albeit rare, resulted in complications such as hematuria. The spleen, muscles, skin, peritoneal and pelvic cavity, brain, and bones were also affected, each exhibiting its unique set of associated complications.

*E. granulosus* (causes cystic echinococcosis) and *E. multilocularis* (causes alveolar echinococcosis) are two different species of parasitic tapeworms with distinct characteristics. The adult size of *E. granulosus* ranges from 2-7 mm, while *E. multilocularis* is slightly larger, measuring between 4-11 mm. The definitive hosts for *E. granulosus* are animals such as dogs, wolves, jackals, and lions, whereas *E. multilocularis *primarily infects foxes. As for intermediate hosts, *E. granulosus* uses sheep and cattle, while *E. multilocularis* targets rodents.

In terms of prevalence, *E. granulosus* is common, but *E. multilocularis* is considered uncommon. The mode of infection for *E. granulosus* typically involves contact with infected dogs. In contrast, infection with* E. multilocularis *can occur through contact with fur from infected foxes or ingestion of contaminated wild berries.

The geographical distribution of the two diseases varies significantly. Alveolar echinococcosis is confined to the Northern Hemisphere, particularly in endemic areas like China and continental Europe. Conversely, hydatid disease is found worldwide, though its prevalence differs between countries.

When untreated, *E. granulosus *has an unusual mortality rate of less than 5% over 10 years, mainly due to anaphylaxis or complicated cysts. In contrast, *E. multilocularis *has a significantly higher untreated mortality rate, ranging from 75% to 100%. The lesion caused by *E. granulosus* typically appears as a fluid-filled unilocular cyst, whereas *E. multilocularis* lesions present as solid masses. Calcification in *E. granulosus* lesions often appears as peripheral calcifications, while *E. multilocularis* lesions feature microcalcification or plaque-like foci [30].

Grossly, alveolar echinococcosis appears as an infiltrative mass with irregular borders, vascular and biliary involvement, and contiguous extension to neighbouring organs and tissues. On US, it appears as a mass mass-like lesion with irregular margins, scattered foci of calcification, central necrosis, and vascular and biliary involvement. On non-contrast CT, the lesion contains hyperattenuating calcification foci and hypoattenuating necrosis and parasitic tissue regions. On contrast-enhanced CT, the lesion shows no substantial enhancement and peripheral fibroinflammatory components with slight but delayed enhancement. On T1 weighted MRI, it appears as a heterogeneous mass with irregular margins and a necrotic centre that exhibits low to intermediate signal intensity.

In comparison, a hydatid cyst grossly is a well-defined cystic or multicystic mass. On US, it appears as a well-defined cystic or multicystic mass containing membranes or septa and hydatid sand; calcification of the cyst wall, matrix, or both. On non-contrast CT, it appears as a well-defined cystic or multi-cystic mass with calcification of the cyst wall, internal septa, or both. The lesion shows no significant enhancement on the contrast-enhanced CT scan. On T1 weighted MRI, it appears as a well-defined, low-signal-intensity cystic or multicystic mass with a low-signal-intensity rim [31].

The limitations of our study warrant consideration for future research endeavours. Firstly, our study was conducted at a single centre, which inherently introduces the potential for bias. Additionally, the relatively small sample size in a single centre, utilized in our study raises concerns about statistical power and results should be further validated with data in a larger population. Furthermore, the age range of our participants was restricted to those above 18 years old, leaving the prevalence and distribution of the phenomenon among younger populations unexplored. Therefore, to enhance the reliability and applicability of our findings, future studies should seek to validate our results using data from larger, more diverse populations encompassing a wider age range.

## Conclusions

Hydatid disease is a systemic condition that can potentially impact any part of the body, with the liver being the most commonly affected organ. Distinctive imaging patterns often highlight its presence, revealing diagnostic features such as peripheral calcifications, internal septations, dense calcifications, daughter cysts, and floating membranes. The disease can lead to various local and systemic complications, including hydatidosis, cyst rupture, rupture into the biliary tree, compression of the biliary system and infection. Recognizing the characteristic appearances and atypical presentations of hydatid disease can facilitate timely, accurate, and efficient diagnoses and treatment of the condition.
